# Prediction of glycosylation sites using random forests

**DOI:** 10.1186/1471-2105-9-500

**Published:** 2008-11-27

**Authors:** Stephen E Hamby, Jonathan D Hirst

**Affiliations:** 1 School of Chemistry, University of Nottingham, University Park, Nottingham NG7 2RD, UK

## Abstract

**Background:**

Post translational modifications (PTMs) occur in the vast majority of proteins and are essential for function. Prediction of the sequence location of PTMs enhances the functional characterisation of proteins. Glycosylation is one type of PTM, and is implicated in protein folding, transport and function.

**Results:**

We use the random forest algorithm and pairwise patterns to predict glycosylation sites. We identify pairwise patterns surrounding glycosylation sites and use an odds ratio to weight their propensity of association with modified residues. Our prediction program, GPP (glycosylation prediction program), predicts glycosylation sites with an accuracy of 90.8% for Ser sites, 92.0% for Thr sites and 92.8% for Asn sites. This is significantly better than current glycosylation predictors. We use the trepan algorithm to extract a set of comprehensible rules from GPP, which provide biological insight into all three major glycosylation types.

**Conclusion:**

We have created an accurate predictor of glycosylation sites and used this to extract comprehensible rules about the glycosylation process. GPP is available online at .

## Background

Most proteins do not perform their function without undergoing some form of post translational modification (PTM) [1]. PTMs occur after the mRNA has been translated into peptide sequence and the polypeptide has begun to fold [2-4]. The importance of PTMs in protein function makes their characterisation of particular interest [2-4]. Accurate prediction, using computational methods, of sites in a protein sequence where PTM occurs would facilitate protein annotation and would contribute to efforts in functional genomics.

Glycosylation [2-4], a common PTM, plays a role in protein folding, transport and half-life, as well as being involved in cell-cell interactions and antigenicity. Glycosylation is an enzymatic process, with the exception of glycation, and involves the addition of sugars to the protein to build up glycan chains. There are four types of glycosylation: N-linked, O-linked, C-mannosylation and GPI (glycophosphatidyl-inositol) anchor attachment. C-mannosylation involves the addition of α-mannopyranosyl to the indole of tryptophan. GPI anchors concern membrane anchoring of a protein by the addition of GPI near the C-terminus. N-linked and O-linked glycosylation are the most common and this study focuses on these modifications.

N-linked glycosylation consists of the addition of a pre-assembled glycan chain to Asn. This occurs co-translationally and influences protein folding. After its addition, the glycan chain undergoes a maturation process, which can produce a glycan of the high mannose, hybrid or complex types. The sequence motif Asn-Xxx-Ser/Thr [5], or in some rare cases Asn-Xxx-Cys, where Xxx is any amino acid except Pro, is required for N-glycosylation, although not sufficient on its own. O-linked glycosylation consists of the stepwise build-up of various sugars on Ser or Thr residues. O-glycosylation has no known consensus sequence [5]. However, Pro is often present around O-glycosylation sites [6] and O-glycosylation occurs more often in the β-strands of proteins [5].

Several glycosylation predictors have been produced [7-10]. Whilst these are not directly comparable, due to development on different datasets, the best predictors appear to be NetOglyc 3.1 [11], which is reported to predict correctly 76% of glycosylated residues and 93% of non-glycosylated residues, and Oglyc [10] with a reported accuracy of 85% correctly classified instances. NetOglyc uses both sequence and predicted structural information (predictions of secondary structure and accessible surface area) to train a back propagation neural network. Oglyc uses support vector machines trained on a combination of physical properties of amino acids and a binary representation of the sequence. In this study we attempt to improve the prediction of glycosylation sites, using a new machine learning algorithm well suited to prediction from protein sequence data.

The random forest algorithm [12] is based on decision trees. A decision tree consists of paths and nodes, with each node using a rule to decide between two or more paths. A rule is typically of the form 'If A then do B', where A is a condition relating to the descriptors of the input data and B is a step on the path through the trees. The last rule gives the classification of the input data example. Several decision trees are developed using a random selection of inputs and random feature selection at each node to grow the trees. The trees then vote on the class for a given input. There is no previous research into predicting glycosylation using random forests, although the algorithm has been widely used, including for prediction of protein-protein interactions [13,14], for analysis of microarray data [15] and identification [16] and prediction [17] of the function of SNPs (single nucleotide polymorphisms). The algorithm has been used for prediction of protein structure from NMR data [18] and amino acid sequence [19]. The random forest algorithm has several features [15], which make it suitable for applications such as the prediction of glycosylation sites. It can be used on a mixture of discrete and continuous descriptors, to classify binary or multi-class data sets and can cope with datasets where there are more variables than observations. The algorithm does not over-fit and continues to be successful, even when there is a large amount of noise in the data.

However, the models generated by random forest can be challenging to interpret. Therefore, we have employed trepan [20], an algorithm originally designed to allow the comprehension of neural networks. It has been adapted for use with other machine learning algorithms [21]. Trepan uses the machine learning algorithm as an "Oracle". By querying the Oracle with the training data and its own generated examples, trepan induces a decision tree using *m *of *n *rules (see methods), thus giving a comprehensible picture of an otherwise opaque machine learning algorithm.

In this paper, using the database of glycosylation sites OGLYCBASE version 6.00 [22], we analyse the amino acid frequencies around glycosylation sites. Using the O-unique dataset  we apply the random forest algorithm implemented in weka [23], combined with information about pairwise patterns, to predict the location of glycosylation sites in a given protein. Pairwise pattern information has previously been used for protein sequence analysis: for example, to predict whether a coiled coil region adopts a leucine zipper structure [24] and to assist in the prediction of protein secondary structure from amino acid sequence [25]. We also experiment with the addition of predicted secondary structure, predicted surface accessibility, and hydrophobicity of the amino acids in an effort to increase the prediction accuracy. Our prediction program is known as GPP (glycosylation prediction program) and is available on-line at: . We would like to interpret the models for the random forest algorithm, and thus gain some biological insight into glycosylation. Whilst random forest produces individual rules that are human readable, in the case of GPP for each of the three types of glycosylation there are ten models of ten trees each. There are redundancies and potentially even conflicts between the different models. We aggregate these models into a single decision tree using the trepan algorithm [20], providing clear rules for each glycosylation type.

## Results and discussion

### Frequency Analysis

We conduct the frequency analysis using the OGLYCBASE dataset. This was used, rather than O-unique, because it has a greater volume and range of sequences allowing statistically significant differences to the background to be more visible. There is also a wider range of sequences than O-unique and it is useful to observe whether there are trends across the whole spectrum of glycol-proteins i.e. is our method likely to be useful for predicting more than just the mammalian glycosylation sites found in O-unique. The consensus sequence for Asn glycosylation is clearly exhibited in the frequency table (Table 1 and Additional file 1). The only amino acids in evidence at the +2 position are Ser, Thr and Cys, with low numbers of Pro at the +1 position. At the -6 position there is an increase in Asp and at the -5 position there is a significant increase in Met. Met is hydrophobic in nature, and is the only such amino acid to be increased around glycosylated Asn residues. At the -2 position Gln is significantly increased. Cys is increased at the +3 position, indicating that Cys assists glycosylation at this position. There is an increase in Pro at the +4 position, which is perhaps surprising, as Pro disfavours glycosylation when found at +1 in almost all cases [5]. It may be that Pro helps create a structural conformation favourable for glycosylation when found at this position.

**Table 1:** Frequencies of selected amino acids surrounding modified Asn residues

position	-7	-6	-5	-4	-3	-2	-1	1	2	3	4	5	6	7
C	12	9	12	12	10	4	5	11	5	**17**	11	8	16	9
D	13	**23**	10	8	9	13	7	8	*0*	14	6	17	12	19
M	13	2	**10**	6	3	5	3	5	0	6	4	5	2	4
P	13	22	15	20	7	15	11	*1*	*0*	6	**31**	18	18	14
Q	9	10	10	16	15	**21**	7	8	*1*	11	13	16	9	11
S	20	22	25	16	16	14	24	23	**102**	32	23	28	11	32
T	14	26	15	23	17	17	15	17	**151**	16	16	13	19	22

Frequency is reported as the number of occurrences in the set of 261 instances of modified Asn residues. Statistically significant increases over the expected frequencies are represented in bold; significant decreases are represented by italics. The full table containing all amino acids is included in additional file 1.

Around modified Ser residues there is known to be an abundance of Pro, Ser and Thr and the frequency analysis (Table 2 and Additional file 2) shows increases of Ser and Thr across the sequence window and increases in Pro at positions -6, -3, -1, 2, 3 and 4. Of those positions where Pro is increased, -1 and +3 present the greatest increases. There is an increase in Ala around the glycosylation site at position -1 perhaps suggesting small amino acids are preferred here. There is also a decrease in Phe at this position. Leu is decreased at -6, -2, +2, and +7, and Lys at +3 and +4. This suggests that these amino acids may have an unfavourable effect on glycosylation.

**Table 2:** Frequencies of selected amino acids surrounding modified Ser residues

position	-7	-6	-5	-4	-3	-2	-1	1	2	3	4	5	6	7
A	22	36	24	36	29	30	**37**	31	34	21	26	30	25	19
D	*6*	12	6	18	14	10	6	4	*3*	10	11	8	5	*5*
E	15	19	22	19	23	24	8	16	10	13	10	11	9	*6*
G	19	26	30	17	20	22	27	40	27	27	16	23	19	41
P	31	**38**	35	35	**41**	34	**46**	28	**40**	**51**	**42**	34	32	35
S	**56**	42	43	**54**	**53**	**56**	47	48	**60**	43	**56**	41	**48**	**49**
T	**58**	**43**	**46**	**41**	**48**	34	**62**	**61**	**48**	**50**	**58**	**51**	**51**	**47**

Frequency is reported as the number of occurrences in the set of 388 instances of modified Ser residues. Statistically significant increases over the expected frequencies are represented in bold; significant decreases are represented by italics. The full table containing all amino acids is included in additional file 2.

Modified Thr residues (Table 3 and Additional file 3) exhibit elevations in Thr at all positions except +7 and Pro at all odd numbered positions. There is an increase in Ser at the -1 position. This suggests that where Thr and Ser glycosylation sites are clustered together, they are almost always consecutive in sequence. Pro is particularly increased at the +3 position, suggesting this is important for glycosylation, as was shown by others [6]. There is a decrease in Ile at position -1 and an increase at -2. Gly is increased downstream at positions -5 and -2, and upstream at positions +1, +4 and +7. Gly is also decreased at -3 and +3. Gln is decreased at the -1 position, as is Lys, which is also decreased at -2, and +1, 2 and 3. There is a general decrease in Leu around the glycosylation site, particularly at the -1 and +1 positions. Arg is decreased at -3, -1 and +3

**Table 3:** Frequencies of selected amino acids surrounding modified Thr residues

position	-7	-6	-5	-4	-3	-2	-1	1	2	3	4	5	6	7
G	52	40	**108**	42	*21*	**122**	32	**101**	40	*16*	**106**	30	37	**112**
I	20	16	16	33	23	**40**	*14*	17	17	13	18	29	14	18
P	**77**	61	**81**	67	**99**	62	**128**	**103**	68	**167**	59	**105**	59	**89**
R	33	22	17	28	*8*	*13*	21	*11*	25	9	15	30	30	16
S	81	67	65	68	74	70	**89**	67	76	74	63	59	52	70
T	**101**	**168**	**96**	**130**	**117**	**115**	**107**	**95**	**118**	**131**	**127**	**95**	**156**	87
W	2	10	4	*1*	2	4	2	2	5	2	3	*0*	*0*	*0*

Frequency is reported as the number of occurrences in the set of 2010 instances of modified Thr residues. Statistically significant increases over the expected frequencies are represented in bold; significant decreases are represented by italics. The full table containing all amino acids is included in additional file 3.

### Pairwise Patterns

The pairwise patterns for each residue type were ranked by weight to identify those most likely to be found around modified residues. These patterns have significant frequencies around unmodified residues, as well as around modified residues. The weights of some patterns are very similar, especially those for Ser, and statistical fluctuations due to the relatively small size of the dataset mean that the rank order of these patterns may not be exact.

Around Asn residues (Table 4) the consensus sequence for Asn glycosylation was visible, with the patterns .......N.T..... (rank 02, weight 3.35) and .......N.S..... (rank 01, weight 4.78) as the top two patterns identified. Other patterns have substantially lower weights indicating the significance of the consensus sequence. Further patterns in the list indicate that Gln at -2 may be significant, as well as Ser, Ala and Arg at various positions. Gln at -2 is also increased in the frequency analysis above and so may be a significant factor. However, there is no significant increase of Ser, Ala and Arg at corresponding positions in the frequency analysis, so it is possible this is only evident as part of a pairwise pattern.

**Table 4:** The 20 most significant patterns for glycosylated residues.

Asn Pattern (weight)	Thr Pattern (weight)	Ser Pattern (weight)
.......N.S..... (4.78)	..........P.T.. (3.39)	.......S..P.... (0.98)
.......N.T..... (3.35)	...T......P.... (2.17)	N......S....... (0.90)
.....Q.N....... (1.78)	..T..P......... (2.14)	.S.....S....... (0.89)
.......N......Q (1.27)	S..T........... (1.74)	.......S......P (0.87)
.......N......S (1.18)	.........S....P (1.57)	.......S.....I. (0.86)
....R..N....... (1.0)	.......T..I.... (1.43)	.......S....P.. (0.83)
......AN....... (1.0)	.......T.....I. (1.39)	....P..S....... (0.82)
..I....N....... (1.08)	...T..P........ (1.25)	.......SA...... (0.80)
.......N.....F. (1.08)	.......T......M (1.25)	.......S.....H. (0.80)
..S....N....... (1.05)	.......T.....P. (1.24)	.......ST...... (0.79)
S......N....... (0.95)	.......TE...... (1.23)	.......S...V... (0.79)
...R...N....... (0.92)	....M..T....... (1.22)	.......S..T.... (0.77)
....F..N....... (0.92)	Q......T....... (1.15)	.......S....R.. (0.77)
...P...N....... (0.89)	.........SP.... (1.11)	.......S.I..... (0.76)
.I.....N....... (0.88)	.M.....T....... (1.0)	......ES....... (0.76)
.......N....A.. (0.88)	....P..T....... (1.0)	......IS....... (0.74)
.......N.....L. (0.88)	......AT....... (1.0)	.......S...P... (0.73)
.....R.N....... (0.86)	.......T..A.... (1.0)	.......S.....A. (0.73)
.......NV...... (0.82)	..........PT... (1.0)	...S...S....... (0.73)
.......N..S.... (0.81)	.............PS (1.0)	.P.....S....... (0.72)

The most significant pattern around Ser is Pro at the +3 position, which is in line with the frequency analysis. Other patterns include Pro Ser, Ile and Thr at various positions indicating that these amino acids may play a prominent role when linked with either Ser or Thr. Many of the patterns around Ser residues have similar weights, although Pro at +3 is markedly more significant.

Whilst no consensus sequence has been shown for Thr, around Thr residues (Table 3) there are correlations between the patterns, which suggest one or more sequence motifs may enhance the propensity for glycosylation. The majority of the patterns in the top 20 contain one of Ile, Thr, Pro or Ser, suggesting that these amino acids favour glycosylation. Given the frequency, and the analysis above (Table 3) it is likely that at least one or more of these amino acids is required for Thr glycosylation. The most prominent pattern is of Pro and Thr at the +3 and +5 positions, respectively. This could indicate either a motif that encourages glycosylation or the importance of the clustering of Ser and Thr glycosylation sites together given the significance of Pro in the neighbourhood of both. There are also several patterns with high significance involving Glu always upstream of the glycosylation site, although no significant increase of this was found in the frequency analysis.

### Prediction accuracy

We measured the prediction accuracy of GPP trained using the pattern weight and sequence only, and using additional structural information. For O-linked glycosylation sites the change in accuracy with additional information was minimal. For N-linked glycosylation an increase in accuracy was observed with the addition of predicted surface accessibility information. There was also a much smaller increase with the addition of predicted secondary structure information (Table 5). The prediction of Thr sites was more accurate than that of Ser sites. The Matthews correlation coefficient, specificity and overall accuracy were higher. However, the sensitivity was higher for the Ser site predictions. This was also the case in for predictions of Ser and Thr carried out with additional information. In comparison to naïve bayes, the prediction by random forest is superior. All predictions by naïve bayes have a substantial loss in sensitivity and a much lower Matthews correlation coefficient.

**Table 5:** Accuracy of prediction of glycosylation sites with random forest and naïve bayes algorithms

	Random Forest				Naïve Bayes			
Dataset (size)	Correctly Classified Instances (%)	Sensitivity (%)	Specificity (%)	Matthews Correlation Coefficient	Correctly Classified Instances (%)	Sensitivity (%)	Specificity (%)	Matthews Correlation Coefficient

Ser	90.8	96.1	88.9	0.81	83.9	64.4	92.6	0.61
Ser + SA	91.1	95.5	89.6	0.82	82.3	60.5	92.3	0.58
Ser + Hydro	89.9	96.4	87.5	0.79	82.7	64.8	90.9	0.59
Ser + SS	91.7	96.3	90.1	0.83	82.4	62.9	91.3	0.58

Thr	92.0	93.6	92.4	0.84	86.8	74.8	93.3	0.70
Thr + SA	91.8	91.4	93.2	0.83	85.8	72.5	93.5	0.69
Thr + Hydro	91.1	91.8	92.2	0.82	85.9	73.0	93.3	0.69
Thr + SS	91.0	91.8	92.1	0.82	87.2	74.7	94.6	0.72

Asn	92.8	96.6	91.8	0.85	90.3	83.8	94.6	0.79
Asn + SA	94.0	95.7	94.3	0.88	89.3	81.9	94.5	0.77
Asn + Hydro	92.4	95.2	91.9	0.84	90.1	82.5	94.8	0.78
Asn + SS	93.2	96.4	92.4	0.86	89.3	79.8	94.9	0.76

Hydro = Hydrophobicity data; SA = predicted surface accessibility; SS = predicted secondary structure.

We first compare the results to the NetOglyc [8], Oglyc [10] and NetNglyc  prediction servers (Table 6). The comparison with O-glycosylation predictors comes with the caveat that they may have been trained and tested with different data, which included differing ratios of positive and negative instances. We also had a slightly different focus than these predictors, in that we do not restrict ourselves to mucin glycosylation sites. For NetOglyc, we use data published in Julenius et al. [11]. The accuracy measures reported did not include correctly classified instances; so we calculated this from the information published. No published results are available for NetNglyc; so we submitted the sequences in the O-unique dataset to the NetNglyc web server and calculated the accuracy measures described above. We also compare predictions for the Asn dataset to a basic pattern search for the consensus sequence carried out by scansite [26]. Li et al. [10] did not give the Matthews correlation coefficient for the Oglyc predictions. Therefore, we calculated it from the reported data and also use the measures of correctly classified instances, sensitivity and specificity for this comparison. We converted the values provided by Li et al. into percentages. Oglyc only report the combined accuracy; separate accuracy information for Ser and Thr was not available. The comparison with Oglyc was carried out against their dataset 2, which produce the best results for their predictor. The GPP predictor has a higher correlation coefficient and sensitivity than NetNglyc. Scansite correctly predicts most positive instances of Asn glycosylation and has a higher sensitivity and specificity than NetNglyc. However, GPP is more accurate and has higher Matthews correlation coefficient, sensitivity and specificity. Our prediction of Thr sites is better in all measures than that of NetOglyc. For Ser prediction our overall accuracy is comparable, although we have a higher Matthews correlation coefficient. NetOglyc has a higher specificity and a lower sensitivity than GPP. There is a higher ratio of negatives to positives in the Ser data set compared to that for Asn and Thr. This affects the pattern weights, bringing them closer together and making it more difficult for the random forest to discriminate between modified and unmodified residues. There are also more types of sugar in more equal proportions in the Ser dataset, creating a more difficult task for the random forest. The Asn dataset does not experience similar effects: its consensus sequence motif is easily picked out (and augmented) by the random forest algorithm. There are no data for separate Ser and Thr predictions available for Oglyc [10]. Their overall prediction accuracy of 87.4% (correctly classified instances) is less than the overall accuracy of GPP, and we also score better in sensitivity and specificity.

**Table 6:** A comparison of the GPP predictor and other glycosylation prediction programs.

	GPP	NetOglyc	NetNglyc	Oglyc	CKSAAP [27]	EnsembleGly [26]	Scan Site
Ser CCI	90.8	91.8	N/A	N/R	83.1	N/R	N/A
Ser Sensitivity	96.1	66.7	N/A	N/R	80.7	N/R	N/A
Ser Specificity	88.9	95.3	N/A	N/R	85.6	N/R	N/A
Ser MCC	0.81	0.62	N/A	N/R	0.671	N/R	N/A

Thr CCI	92.0	84.9	N/A	N/R	81.4	N/R	N/A
Thr Sensitivity	93.6	81.5	N/A	N/R	80.3	N/R	N/A
Thr Specificity	92.4	89.5	N/A	N/R	82.5	N/R	N/A
Thr MCC	0.84	0.67	N/A	N/R	0.63	N/R	N/A

Asn CCI	92.8	N/A	76.7	N/A	N/A	95.0	79.8
Asn Sensitivity	96.6	N/A	43.9	N/A	N/A	98.0	72.7
Asn Specificity	91.8	N/A	95.7	N/A	N/A	77.0^b^	81.9
Asn MCC	0.85	N/A	0.49	N/A	N/A	0.84	0.54

Overall CCI	91.4^a^	88.6^a^	N/A	87.0^a^	N/A	89.0	N/A
Overall Sensitivity	94.9^a^	76.0^a^	N/A	92.0^a^	N/A	59.0	N/A
Overall Specificity	90.7^a^	92.8^a^	N/A	78.0^a^	N/A	68.0^b^	N/A
Overall MCC	0.83^a^	0.66^a^	N/A	0.71^a^	N/A	0.64	N/A

a. combined accuracy for Ser and Thr

b. Specificity for EnsembleGly was calculated as *T*_*p*_/*T*_*p*_+*F*_*p*_. See text for comparison.

N/A = not applicable; N/R = not reported; CCI = % correctly classified instances; MCC = Matthews Correlation Coefficient

Two more recent predictions servers, EnsembleGly by Caragea et al. [26] and CKSAAP by Chen et al. [27], were published during the completion of this work. Caragea et al. use ensembles of support vector machines to predict O- and N-linked glycosylation sites. Caragea et al. calculate sensitivity as *S*_*n *_= *T*_*p*_/(*T*_*p*_+*F*_*p*_). We convert this measure into a percentage. Calculating this measure for GPP, for Asn prediction, *S*_*n *_= 87.2; for Ser, *S*_*n *_= 81.3; for Thr, *S*_*n *_= 87.5; and for the combined O-linked predictions, *S*_*n *_= 84.4. GPP has a greater Matthews correlation coefficient for both N- and O-linked prediction (only an overall score for O-linked is given). For N-linked sites they have a greater accuracy and sensitivity, but GPP has greater specificity and Matthews correlation coefficient, indicating EnsembleGly has a greater number of false negative predictions. For O-linked sites, GPP scores better for sensitivity, specificity and Matthews correlation coefficient. Chen et al. predict mucin glycosylation sites using k-spaced pairwise patterns and support vector machines. This method has some similarities with our own and the accuracy of the two methods is comparable. However, GPP is more accurate for both Ser and Thr predictions.

### Rule extraction

**Figure 1:**
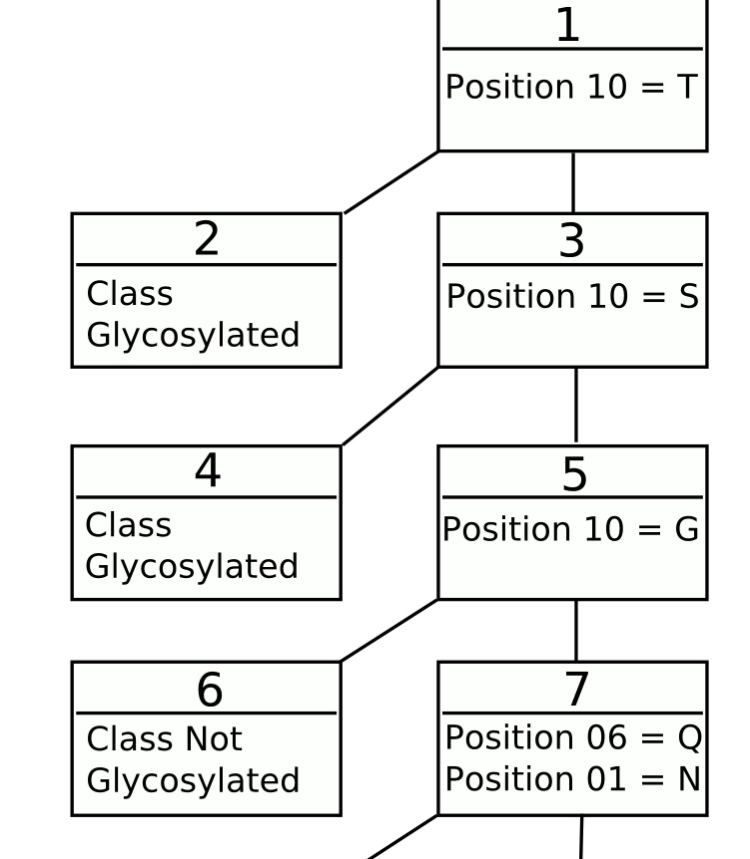
**Asn glycosylation rules**. A subset of the complete decision tree covering all the rules for Asn glycosylation (the complete tree is available as additional file 4). Each node is numbered in the order it was added to the tree. All rules are 1 of A, B...N so only the relevant features are shown. The amino acids are represented using the single letter code and the positions are indicated with respect to a sequence window of length 15, with the target residue at position 08.

**Figure 2:**
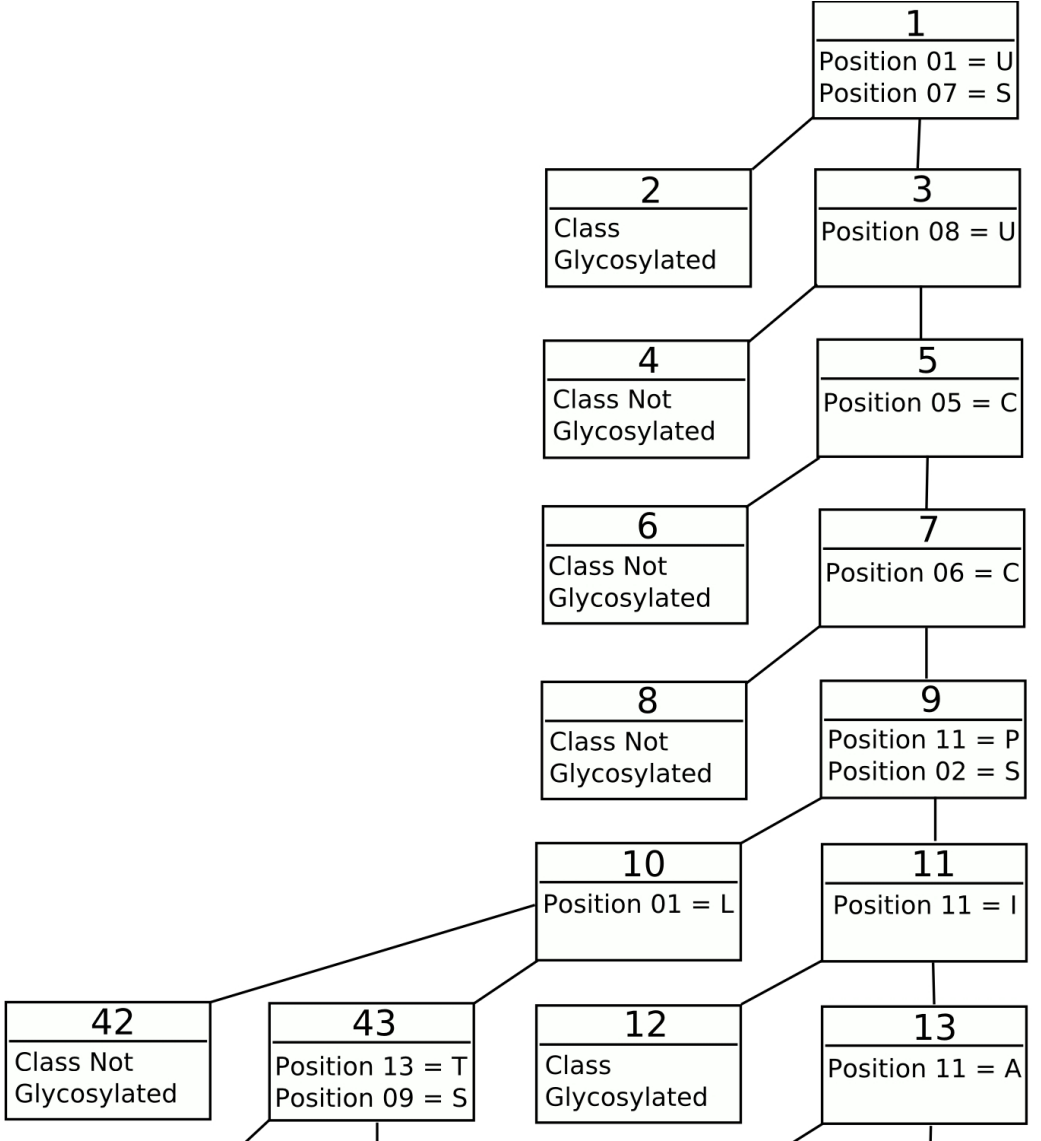
**Thr glycosylation rules**. A subset of the complete decision tree encompassing all the rules produced for Thr glycosylation (the complete tree is available as additional file 5). Each node is numbered in the order it was added to the tree. All rules are 1 of A, B...N so only the relevant features are shown. The amino acids are represented using the single letter code. The amino acids are represented using the single letter code amino acid and the positions are indicated with respect to a sequence window of length 15, with the target residue at position 08.

**Figure 3:**
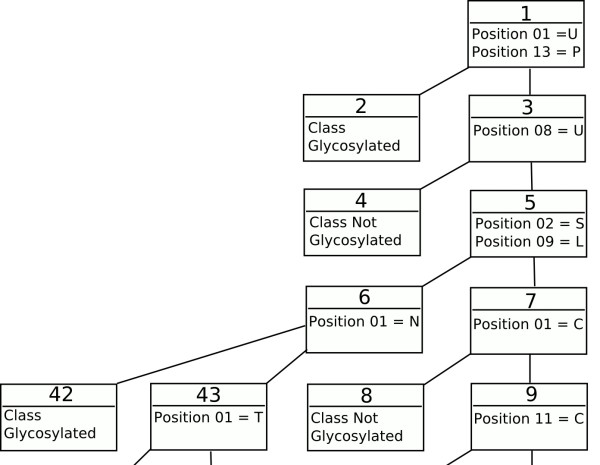
**Ser glycosylation rules**. A subset of the rules produced for Ser (the complete tree is available as additional file 6) showing the importance of the +2 position in glycosylation of Ser. Each node is numbered in the order it was added to the tree. All rules are 1 of A, B...N so only the relevant features are shown. The amino acids are represented using the single letter code and the positions are indicated with respect to a sequence window of length 15, a sequence window of length 15 with the target residue at position 08.

Trepan identifies the consensus motif for Asn glycosylation (figure 1 and additional file 4) as the most prominent rules in the decision tree. However, subsequent rules are somewhat misleading as they allow glycosylation without the consensus sequence being present. This is probably an artefact of the generation of additional data by trepan. This approach is reliant on the distribution of the training data and will highlight patterns additional to the consensus sequence. The tree corresponding to Thr glycosylation (figure 2 and additional file 5) shows features in line with the statistical data. Pro at residue +3 increases glycosylation when accompanied by a Ser or Thr. The end of the sequence seems to be given undue importance. However, other rules are in line with the frequency analysis. Cys seems to strongly discourage glycosylation, whilst Ser, Thr and Pro encourage it when accompanied by various other amino acids. Some rules may be inexact, due to the limited data in O-unique that trepan can base its derived examples on. This is also true for the Ser tree (figure 3 and additional file 6). The tree for Ser is similar to the one for Thr, although more complicated. Once again the end of the sequence is implicated as is the presence of Pro at various positions. Cys again seems to block glycosylation, whilst Ser, Thr, Glu, and Pro all encourage it when present at various positions along the sequence, especially in conjunction.

## Conclusion

The random forest algorithm was used to predict glycosylation sites, based on pairwise sequence patterns and the amino acid sequence. The program improved over the best prediction programs currently available, with significant increases in accuracy for the prediction of Thr and Asn glycosylation sites. Neither the addition of structural data, hydrophobicity information nor surface accessibility data improved the prediction accuracy of O-linked glycosylation, although N-linked glycosylation prediction is improved by the addition of surface accessibility data. However, it may be possible to improve prediction accuracy further through the inclusion of information on protein disorder and information on the orientation of membrane proteins. It may also be possible to increase accuracy by extending the initial data set, or by considering separately proteins whose PTM is catalysed by the same enzyme. Another option would be to produce prediction programs for each specific glycan type, or to classify each glycosylation site by type of glycan after prediction. Our use of the trepan algorithm allows us to extract comprehensible rules describing features characteristic of a glycosylation site.

## Methods

### The dataset

The data for frequency analysis is taken from OGLYCBASE 6.00 [22] available online from . The OGLYCBASE database contains both experimentally verified and putative instances of N-, O-, and C-linked glycosylation sites. It comprises 242 protein sequences and 2413 verified glycosylation sites. The C-mannosylation data were not considered in our investigations, because there are too few experimentally verified sites in the dataset. Although several enzymes catalyse the attachment of a glycan to Ser and Thr, we have considered all cases in our dataset, with the expectation that the sequence patterns surrounding the glycosylated residue may nevertheless be similar, or at the very least that the machine learning algorithms may be able to detect and learn different sets of patterns within the dataset. For training and evaluation of GPP by ten-fold cross-validation, we use the O-unique dataset. This is a subset of OGLYCBASE and was used for the training of NetOGlyc. It contains only mammalian proteins and is non-redundant. Our predictions were based on only those glycosylation sites that have been experimentally verified. Unverified sites can sometimes be unreliable and false results may confound the predictions. The information retained from the database consisted of the sequence, database reference and the location in the sequence of the modified residues that have been experimentally verified. The both datasets were then split into three, according to whether the modified residues are Ser, Thr or Asn. Within the O-unique dataset, the Ser data set contains 1219 instances (395 positive and 824 negative), the Thr dataset contains 1068 instances (370 positive and 698 negatives) and the Asn dataset contains 589 instances (200 positive and 389 negatives). After removing duplicate sequence windows from the OGLYCBASE datasets, the Ser dataset contains 7285 instances (349 positive 6936 negative) The Thr dataset contains 6389 instances (695 positive and 5694 negative) and the Asn dataset contains 3508 instances (261 positives and 3247 negatives). Each instance was considered as the potentially modified residue and seven residues on either side, to give a 15 amino acid sequence window. This choice of window size was based on previous work [10], providing reasonable computational tractability in determining pairwise patterns in the data, and still maintaining sufficient information to predict glycosylation site location. In this work, we use the single letter code to represent the amino acids in a categorical fashion. The weight of each instance derived from the patterns was represented by a numerical attribute. The random forest algorithm can develop trees using a mixture of discrete and continuous data. So no additional processing of the data was necessary before presenting the data to weka to train the random forest algorithm.

### Frequency Analysis

After removing all duplicate sequence windows of size 15 from OGLYCBASE, we determined the frequency of each type of amino acid at each positioning the window. This was carried out for both modified and unmodified sites for the Ser, Thr, and Asn datasets and on all of these combined. The frequencies of the modified sites were considered to be significant if the difference between the expected frequency and the actual frequency was greater than 3*σ*, where *σ *is the standard deviation. The expected frequency of the residue *i *at position *j *was calculated as:

(1) *E*_*ij *_= *F*_*ij *_*N*_*mod*_/*N*_*unmod*_

where *N*_*mod *_is the number of sequence windows centred on modified residues, *N*_*unmod *_is the number of windows centred on unmodified residues and *F*_*ij *_is the frequency of occurrence of residues *i *at position *j *in the unmodified windows. The standard deviation was estimated assuming a binomial distribution. We focus on frequent patterns in modified sequences, as there is no obvious reason to anticipate that strong negative sequence motifs have evolved to evade recognition by enzymes catalysing glycosylation.

The frequency of each possible unique pairwise arrangement of amino acids in the window was calculated. Patterns below a given frequency threshold were excluded from the final pattern set. To optimise the threshold for pattern exclusion a single data set was prepared for each residue type consisting of all positives and an equal number of negatives; the threshold was increased incrementally and each resulting pattern set was used for prediction. The thresholds that produced the best accuracy were used in the final prediction program. This gave thresholds of 22 for Asn, 31 for Ser and 15 for Thr.

Each pattern is given a weighting, to provide a measure of the probability that a sequence containing that pattern is a member of the modified class. For a pattern *x*, the pattern weight *W*_*x *_is calculated as *F*_*m*_/*F*_*n *_where *F*_*m *_is the frequency of modified sequence windows in which pattern *x *occurs and *F*_*n *_is the frequency of unmodified windows in which this pattern occurs. Each sequence window is compared against all of the significant patterns for that type of glycosylation site. Based on the patterns found, the sequence is given a pattern weight *W*^*seq*^.:

(2) $$W^{seq}=\sum_{x=1}^{k}W_{x}/k$$

where *W*_*x *_is the weight of pattern *x*, and *k *is the number of patterns found in the sequence. The weight and the sequence window are presented in the form of a string of letters (the single letter code for amino acid representation) comprising the sequence window and a numerical value (the weight) making use of the capability of weka [23] to handle a mixture of continuous and categorical data.

**Figure 4:**
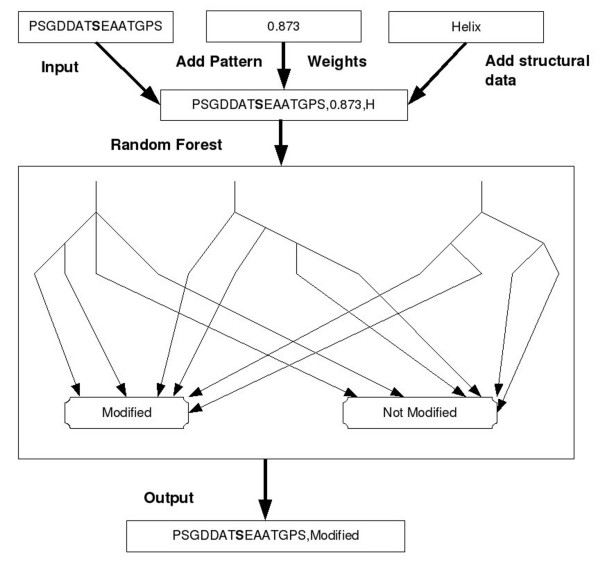
**The flow of data through the prediction program**.

Predicted secondary structure information was combined with the pairwise pattern information described above. The program PsiPred [29] was used to predict the secondary structure of the residue at the centre of each sequence window and this was then placed after the window sequence and the corresponding weight from pattern analysis. The surface accessibility was predicted, using the SABLE program [30], as a number between 0 and 100, with 0 representing fully buried and 100 fully exposed. The data obtained from SABLE were added to the central residue of the corresponding instances in the training data. The hydrophobicity value of each central residue was added to the corresponding instance in the training data. These hydrophobicity values were taken from the literature [31]. The data flow through the prediction program is shown in figure 4.

### Training the prediction program

**Figure 5:**
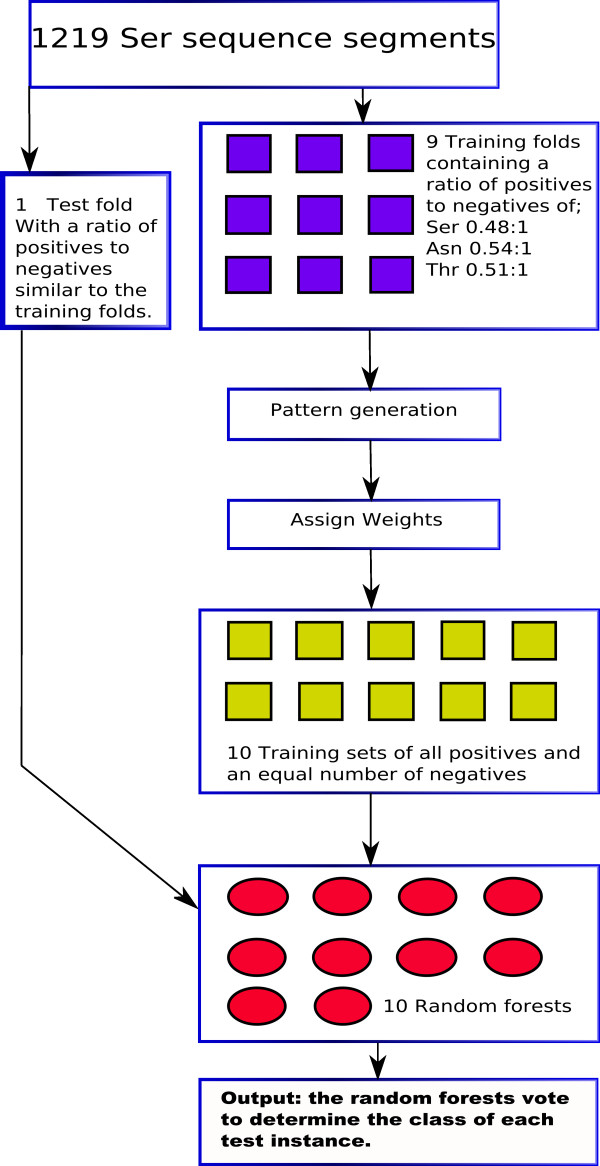
**The cross-validation of the GPP prediction program, illustrated for the Ser dataset**. This procedure is repeated 10 times with each fold in turn being used as the test set in order to conduct a cross validation. The 10 training sets are drawn from the sum of the 9 folds of training data and are used to train 10 random forests.

Training of the prediction program has two main components. Firstly, a set of patterns is generated from the training data for each of the three types of glycosylation site. This is then used to provide a weighting to each instance in the dataset. Secondly, the random forest is trained on the data and associated weights. Multiple random forests (ten in this work) are trained, with each voting to determine the class of each test instance. Each of the random forests was trained using a data set comprising all positive instances from the cross validation fold and an equal number of randomly chosen negative instances, this dataset being generated from the training data. We use multiple forests to allow for as complete as possible representation of negative instances in the training data without the negatives completely overwhelming the positives in the dataset. The pattern sets were created from the entire training data within a cross validation fold. This entire procedure is summarised in figure 5. The accuracy of the prediction was evaluated by cross validation. The data were divided randomly into ten sections and the above training procedure was carried out using nine of these, the tenth providing a test set using all instances. This was repeated ten times on each occasion with a different section of the data acting as the test set. The measures of accuracy used to assess the prediction program are as follows. Sensitivity, expressed as a percentage, is calculated as *T*_*P*_/(*T*_*P*_+*F*_*N*_) × 100, where *T*_*P *_is the number of true positive predictions and *F*_*N *_is the number of false negative predictions. Specificity, expressed as a percentage, is calculated as *T*_*N*_/(*F*_*P*_+*T*_*N*_) × 100, where *T*_*N *_is the number of true negative predictions and *F*_*P *_is the number of false positive predictions. The number of correctly classified instances is given as a percentage. We use the Matthews correlation coefficient [32] to compare the accuracy of our prediction program with that of the NetNglyc  and NetOglyc [11] glycosylation predictors. The Matthews correlation coefficient is calculated as follows:

(3) $$MCC=\frac{\left(T_{P}T_{N})-(F_{P}F_{N}\right)}{\sqrt{\left(T_{N}+F_{N})(T_{N}+F_{P})(T_{P}+F_{N})(T_{P}+F_{P}\right)}}$$

We use the number of correctly classified instances, the sensitivity and the specificity to compare our work with Oglyc [10].

In order to test the significance of the differences between the different methods of prediction, a paired *t *test [33] was conducted on 30 duplicate experiments for pairs of methods. Given a set of results *X*_*i *_from method *A *and a set of results *Y*_*i *_from method *B*, each containing *n *data points, *t *is calculated as:

(4) $$t=\left(\bar{X}-\bar{Y}\right)\sqrt{\frac{\left(n\left(n-\text{1}\right)\right)}{\sum_{i=\text{1}}^{n}{\left({\hat{X}}_{i}-{\hat{Y}}_{i}\right)}^{\text{2}}}}$$

where $\bar{X}$ is the mean of *X *and $\bar{Y}$ is the mean of *Y*, ${\hat{X}}_{i}=\left(X_{i}-\bar{X}\right)$; ${\hat{Y}}_{i}=\left(Y_{i}-\bar{Y}\right)$ and *p *is the probability of obtaining a value as large or larger than the observed *t*. If *p *is below 0.05 then the difference of means is significant at the 5% level. The *t *test was calculated using the R statistics package.

For the purposes of comparison we also conducted the above procedure substituting the naïve bayes algorithm for random forest. The naïve bayes algorithm is based on Bayes rule, which states that for a given input vector *x*_1_......*X*_*n *_the probability of observing a class *M *is

(5) *P*(*M*|*x*_1_,.....,*x*_*n*_) = *P*(*x*_1_,.....,*x*_*n*_|*M*)*P*(*M*)/*P*(*x*_1_,.....,*x*_*n*_)

Whilst it is theoretically possible to estimate the probability for each class *M*, in practice the conditional probabilities are not usually known and must be estimated from the data. For this reason the naïve bayes algorithm makes the assumption that the conditional probabilities are independent given the class in order to simplify equation 5 to:

(6) *P*(*x*_*i*_|*M*) = (*P*(*x*_1_)),...,(*P*(*x*_*n*_))

Although this is a rough approximation of the probability for a given class, the naïve bayes classifier has proven to be reasonably robust, because it only matters that the true class receives the highest probability, not that the probability itself is correct. We used the implementation of naïve bayes in weka [23]. As a further comparison we also carried out a basic pattern search using scansite [28], which classifies as positive all sites that have the consensus sequence. This was performed on the entirety of O-unique, since no training is required for scansite.

### Extraction of Rules

Trepan is a method originally used to extract comprehensible rules from neural networks. Trepan uses an oracle function to represent the network and derives a decision tree from the classifications made by the oracle function. However, it can be used for rule extraction from any method that performs binary classification. We use here a modified version of trepan implemented in Matlab [21], with GPP as the oracle function. Thus, we derive a decision tree based on the classification by GPP of the training data, and additional examples created by trepan. The additional examples are based on the distribution of the attributes in the training data and they ensure a pre-set minimum number of examples reach each node in the tree. The splitting test at each node is an *m *of *n *test. For each node in the tree there are *n *features. If *m *of these features are evident in a given instance, this instance is deemed to satisfy the *m *of *n *rule for this node. In practice here we find rules only with *m *= 1 and *n *< 3, i.e. simple predicates involving one or two possibilities. Nodes of the tree are expanded based on a priority calculated as the number of examples misclassified by the node. Those with highest priority are expanded first, since they have the most potential to increase the accuracy of the tree.

## Authors' contributions

SEH conducted the experiments and wrote the manuscript. JDH conceived the study and assisted in writing the manuscript. All authors read and approved the final manuscript for publication.

## Supplementary Material

Additional file 1 **The frequencies of amino acids surrounding modified and unmodified Asn residues**. Full tables containing all amino acids (as opposed to the partial data presented in Table 1 of the paper). Statistically significant increases over the expected frequencies are starred; significant decreases are labelled with a 'd'. Click here for file

Additional file 2 **The frequencies of amino acids surrounding modified and unmodified Ser residues**. Full table containing all amino acids (as opposed to the partial data presented in Table 2 of the paper). Statistically significant increases over the expected frequencies are starred; significant decreases are labelled with a 'd'. Click here for file

Additional file 3 **The frequencies of amino acids surrounding modified and unmodified Thr residues**. Full table containing all amino acids (as opposed to the partial data presented in Table 3 of the paper). Statistically significant increases over the expected frequencies are starred; significant decreases are labelled with a 'd'. Click here for file

Additional file 4 **The complete decision tree covering all the rules for Asn glycosylation**. Full decision tree, extending the subset shown in Figure 1 in the paper. Click here for file

Additional file 5 **The complete decision tree covering all the rules for Thr glycosylation**. Full decision tree, extending the subset shown in Figure 2 in the paper. Click here for file

Additional file 6 **The complete decision tree covering all the rules for Ser glycosylation**. Full decision tree, extending the subset shown in Figure 3 in the paper. Click here for file
