# Supplementary figures and images for: Prediction of glycosylation sites using random forests
Source: BMC Bioinformatics. 2008 Nov 27;9:500. doi: 10.1186/1471-2105-9-500 (PMC2651179; doi:10.1186/1471-2105-9-500)

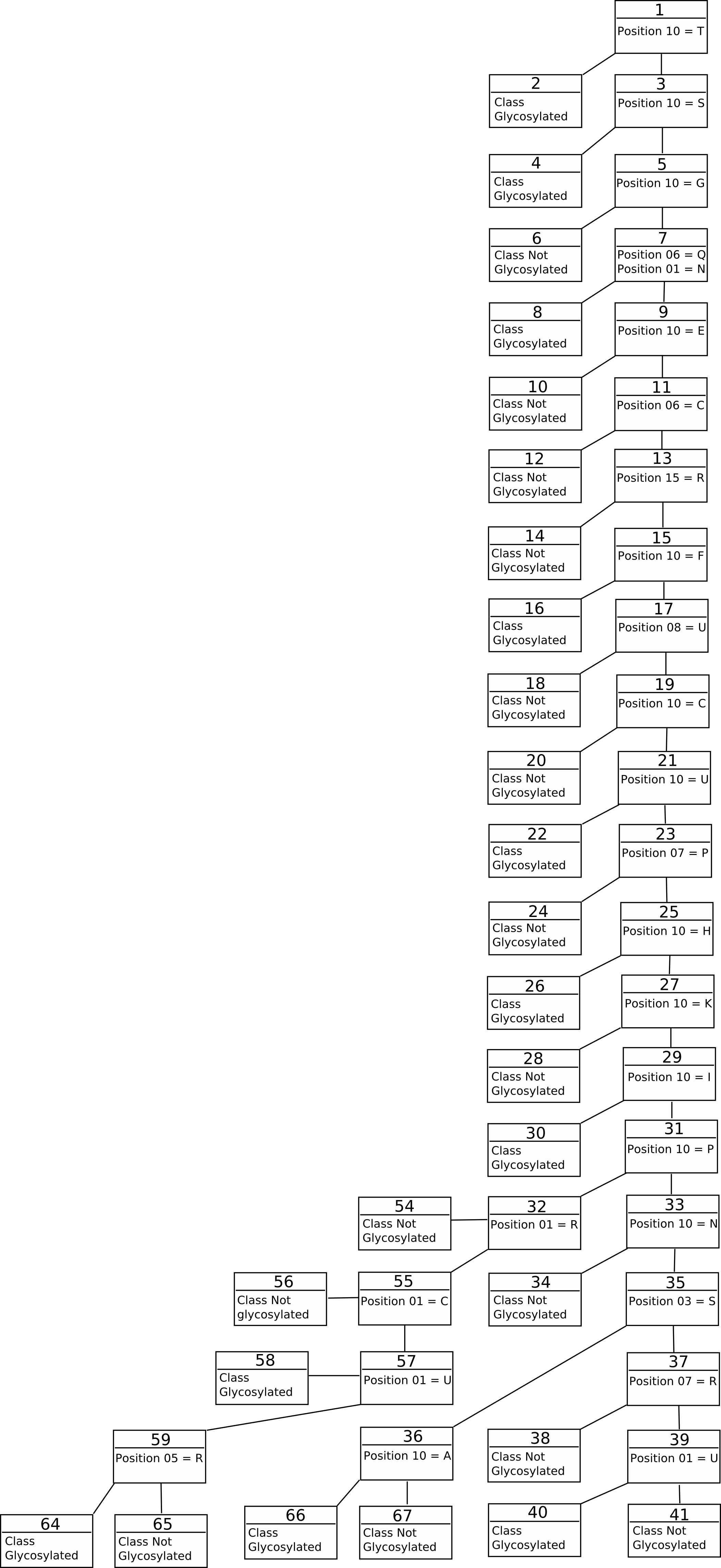

Supplement: Additional file 4 — The complete decision tree covering all the rules for Asn glycosylation. Full decision tree, extending the subset shown in Figure 1 in the paper. [file 1471-2105-9-500-S4.png]

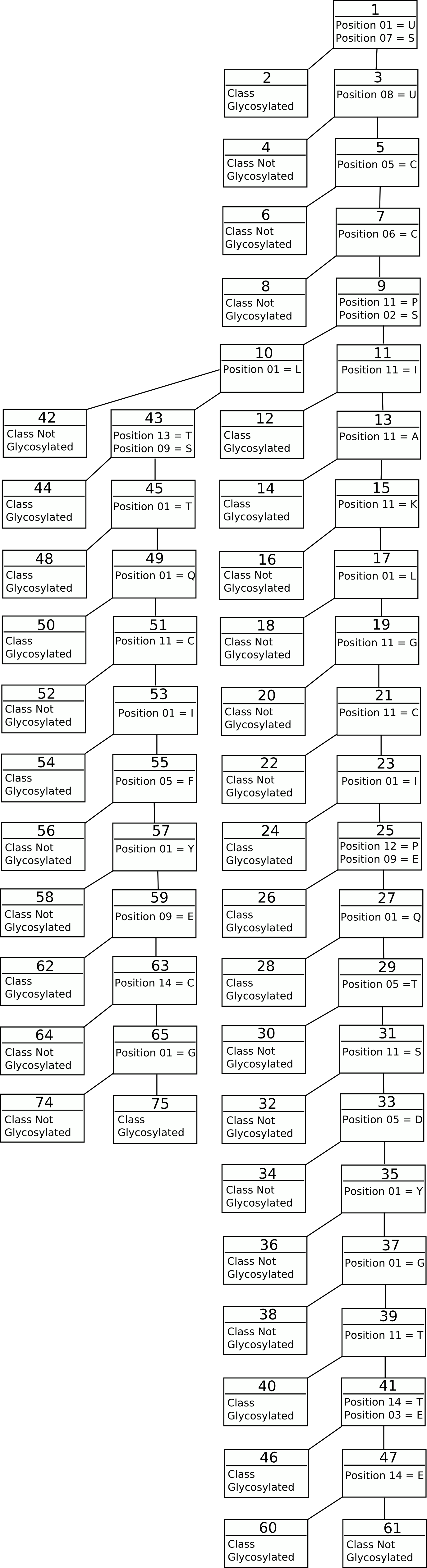

Supplement: Additional file 5 — The complete decision tree covering all the rules for Thr glycosylation. Full decision tree, extending the subset shown in Figure 2 in the paper. [file 1471-2105-9-500-S5.png]

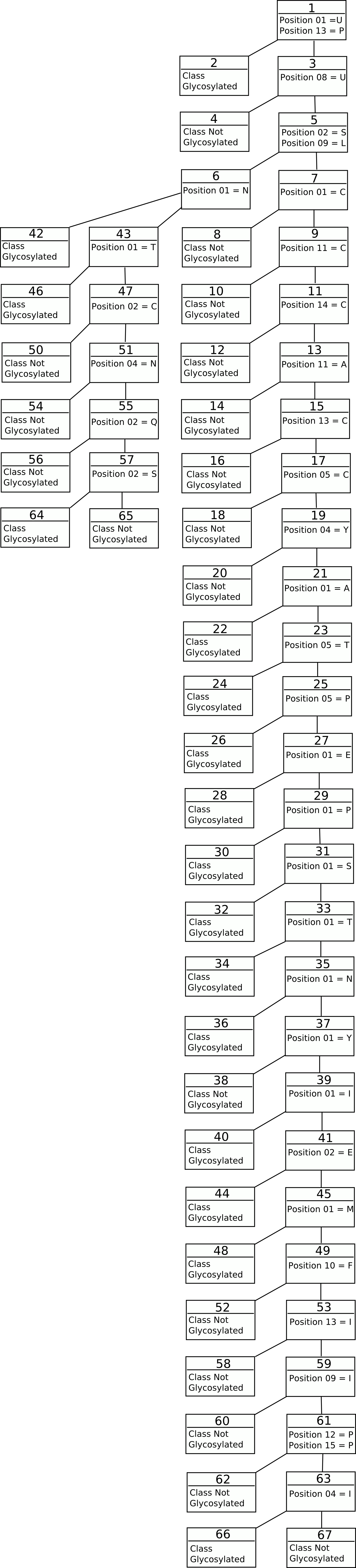

Supplement: Additional file 6 — The complete decision tree covering all the rules for Ser glycosylation. Full decision tree, extending the subset shown in Figure 3 in the paper. [file 1471-2105-9-500-S6.png]
